# Dyadic interaction enhances sensitivity and shifts decision criterion in a change detection task

**DOI:** 10.3389/fcogn.2026.1827978

**Published:** 2026-08-03

**Authors:** Tatsuru Kawachi, Yousuke Kawachi

**Affiliations:** Department of Psychology, Graduate School of Arts and Letters, Tohoku University, Sendai, Japan

**Keywords:** change detection, decision criterion, decision-making, dyads, response bias, sensitivity, signal detection theory

## Abstract

**Introduction:**

Can two-person decision-making change performance in detecting a change in the scene? Observers sometimes fail to detect visual changes that occur during a coincident visual transient or other disruption, a phenomenon known as change blindness. Although change blindness has been extensively studied, relatively little is known about whether collaborative decision-making can reduce such failures. Research on collective decision-making suggests that dyads make more accurate judgments than individuals, and recent findings show that they also adopt a more liberal decision criterion. This study examined whether dyads exhibit differences in sensitivity and decision criteria in a change detection task.

**Methods:**

Participants in a dyad judged individually and collectively whether the pair of flickering images contained a change. We also conducted a follow-up independent repeated decision-making experiment, in which individuals responded twice with a period of time between two judgments, to confirm whether the second judgment without social input differed from the first judgment.

**Results:**

Results from the dyadic decision-making experiment showed that dyads demonstrated enhanced sensitivity to detect changes and were more likely to respond “change present” than better individuals, suggesting the dyadic decisions were not simply based on the judgment of the more sensitive member. Results from the follow-up experiment supported the effect of dyadic decision-making by indicating no difference in sensitivity and decision criterion between first and second judgments.

**Discussion:**

These findings suggest that dyadic decision-making may help reduce the failure of change detection by improving sensitivity and shifting the decision criterion to minimize missed detections.

## 1 Introduction

Detecting changes in the scene is fundamental to human behavior and is especially important for detecting danger. However, even when a substantial change occurs, observers sometimes fail to detect it. This phenomenon, known as change blindness, has been documented across various contexts, including flickering images (Rensink et al., 1997), alternating images with mudsplashes (O'Regan et al., 1999), dynamic video clips with camera pans or cuts (Fitzgerald et al., 2016), and real-world environments (Attwood et al., 2018; Simons and Levin, 1998). Research has extensively investigated individual factors that induce change blindness, showing that detection failures can arise from insufficient attention (e.g., Rensink et al., 1997) or weak visual representation of the original scene (Rensink, 2002; Simons, 2000). More recently, individual differences in detection failures have been linked to visual stability (the capacity to maintain robust representations despite distraction) and visual ability (reflecting low-level perceptual sensitivity) (Andermane et al., 2019). Hence, failures of change detection challenge our intuition about possessing rich and detailed visual representations, and contribute to our understanding of the limitations and mechanisms of visual perception and awareness (Simons and Levin, 1997; Simons and Rensink, 2005).

Researchers have investigated factors that are related to change detection performance. Early research (Rensink et al., 1997) indicated that changes in objects of high interest, referred to as central interest, in a certain scene were detected more quickly than those of low interest, referred to as marginal interest. In a related vein, Werner and Thies (2000) reported that experts in a certain domain detected semantic changes in domain-specific scenes faster than novices. These studies have emphasized the relationship between change detection performance and semantic aspects of changes. Lee et al. (2023) further focused on task demands related to change detection and showed that change detection performance improved when participants engaged in searching for a prechange target actor compared with when they engaged in counting actors or remembering the target actor. Training can also improve change detection performance, although such improvements may not always generalize across tasks (Gaspar et al., 2013; Taylor et al., 2017). However, these factors are often tied to individual observers, specific stimulus properties, or task contexts.

Based on findings regarding change blindness, there is a limit to what individuals can do to avoid missed detections. Rather than attempting to eliminate these individual limitations, a simple solution to overcome failure to detect changes is to increase the number of observers. We often make decisions based on aggregated judgments of multiple individuals, such as when a team of security officers screens baggage at an airport checkpoint. Previous work on collective decision-making has demonstrated that combining information across individuals can improve performance. Building on this line of research, (Bahrami et al. 2010, 2013) showed that dyadic decision-making in perceptual tasks often surpasses individual performance through interaction between observers.

However, the consequences of collective decision-making do not necessarily reflect enhanced perceptual sensitivity alone. Response bias is a key factor in understanding the outcome of a judgment (Peters et al., 2016). Signal detection theory (SDT; Green and Swets, 1966) provides a valuable framework for disentangling these factors, as it allows researchers to independently measure the sensitivity (the ability to detect changes) and the decision criterion (response tendency or bias) from yes–no task data (Green and Swets, 1966; Wixted, 2020). For example, experienced drivers have a more liberal decision criterion (tend to anticipate a potential traffic conflict) and respond more quickly to hazards than novice drivers, despite no difference in sensitivity between groups (Wallis and Horswill, 2007). This example illustrates that response bias can shape behavioral outcomes independently of perceptual sensitivity. Notably, a recent study reported that dyadic decision-making shifts the decision criterion toward a more liberal position and enhances visual sensitivity in a low-level detection task (Kawachi and Kawachi, 2025). Thus, dyadic decision-making may alter change detection performance by modulating both visual sensitivity and decision criterion, although these two components have different implications for detection accuracy and response bias. Still, it remains unclear whether such changes in sensitivity and decision criterion can be found in a dyadic decision-making task requiring higher-order perceptual and cognitive processing than the low-level detection task used in the previous study (Kawachi and Kawachi, 2025).

Taken together, in what aspects can dyadic decision-making change performance in a change detection task? The present study investigated whether dyadic decision-making enhances sensitivity and shifts the decision criterion in a change detection task. In the dyadic decision-making experiment, we compared the sensitivity and decision criterion of dyads with those of the more sensitive member in each pair to examine whether dyadic decisions were made by simply adopting the opinion of the more sensitive member. Based on Kawachi and Kawachi (2025), we hypothesized that dyads would demonstrate greater sensitivity to detect changes and adopt a more liberal decision criterion, tending to respond “change present” more often than more sensitive individuals of each dyad. This means that the fluctuation in sensitivity and decision criterion in the dyad condition reflected the dyadic interaction rather than dependence on the more sensitive member. We also conducted a follow-up independent repeated decision-making experiment to examine whether a factor other than dyadic interaction could account for the results of the dyadic decision-making experiment. In the dyadic decision-making experiment, participants judged individually and collectively, indicating the possibility of behavioral changes due to responding twice. Regarding this individual factor, in the follow-up experiment, we tested whether the second judgment without social input could account for the observed enhancement in sensitivity and shift in the decision criterion. If there were no differences in sensitivity and decision criterion between the first and second judgments, this would help rule out the possibility that the results of the dyadic decision-making experiment were merely attributable to the effect of judging twice.

## 2 Materials and methods

### 2.1 Participants

Twenty-two dyads (44 participants, 20 women; mean age = 19.39, *SD* = 1.46) participated in the dyadic decision-making experiment. Each dyad comprised same-sex friends to minimize the effects of gender stereotypes (Bahrami et al., 2010; Pescetelli et al., 2016) and social hierarchy. The sample size was not determined *a priori*. Instead, we adopted a greater sample size than previous studies (Bahrami et al., 2010; Kawachi and Kawachi, 2025). A *post hoc* sensitivity analysis by G^*^power Version 3.1.9.6 (Faul et al., 2007) revealed that this sample size (*n* = 22) was sufficient to detect a medium (Cohen's *d* = 0.5) to large (Cohen's *d* = 0.8) effect size (Cohen's *d* > 0.67) with a statistical power of over 84% (α = 0.05).

Based on the results of the dyadic decision-making experiment, a follow-up experiment was conducted to examine whether individual second judgments, without social input, differed from the first judgments in sensitivity and decision criterion. In the follow-up experiment, the required sample size was estimated using G^*^power Version 3.1.9.6 (Faul et al., 2007), with power (1—β) set at 0.90, α = 0.05, and Cohen's *d* = 0.67 (medium to large effect size) for a paired *t*-test. The effect size was set based on the results of the dyadic decision-making experiment. Results showed that 26 participants needed to achieve a 90% power at α = 0.05. Because the same stimuli were used in both the dyadic decision-making and follow-up experiments, twenty-seven individuals who had not been recruited in the dyadic decision-making experiment participated in the follow-up experiment, but one participant could not complete the experiment because of an error in the computer program. As a result, the data from 26 participants (14 women; mean age = 21.35, *SD* = 2.66) were used in the analysis.

### 2.2 Apparatus and experimental setup

A desktop personal computer (PC) (XPS720, DELL, Texas, USA) and two LCDs (ZOWIE XL2411; resolution: 1,920 × 1,080 pixels; refresh rate: 60 Hz; BenQ Corporation, Taiwan) were used. The stimuli presentation and experimental procedures were controlled using the Cogent 2,000 and Cogent Graphics toolboxes for MATLAB 2007b (MathWorks, Natick, MA, USA).

Participants were seated side-by-side in a dimly lit room, each viewing the display from a distance of approximately 50 cm. While they could see each other, partitions prevented them from viewing one another's screens in the dyadic decision-making experiment.

### 2.3 Stimuli

Natural scene images from the Change Blindness (CB) database (Sareen et al., 2016) were used as stimuli for this experiment. To lessen the burden of the experiment on participants and reduce task difficulty despite a few flickers, we chose stimuli with the shortest mean reaction times (range = 1.67s-−17.86s, *M* = 7.97, median = 7.11), where shorter reaction times indicated easier change detection. Each stimulus pair comprised an original and a modified image with an added object. Consequently, 96 image pairs (192 images) were used in the dyadic decision-making and follow-up experiments. Each image subtended 30.8° × 23.4° of visual angle.

### 2.4 Procedure

**Figure 1:**
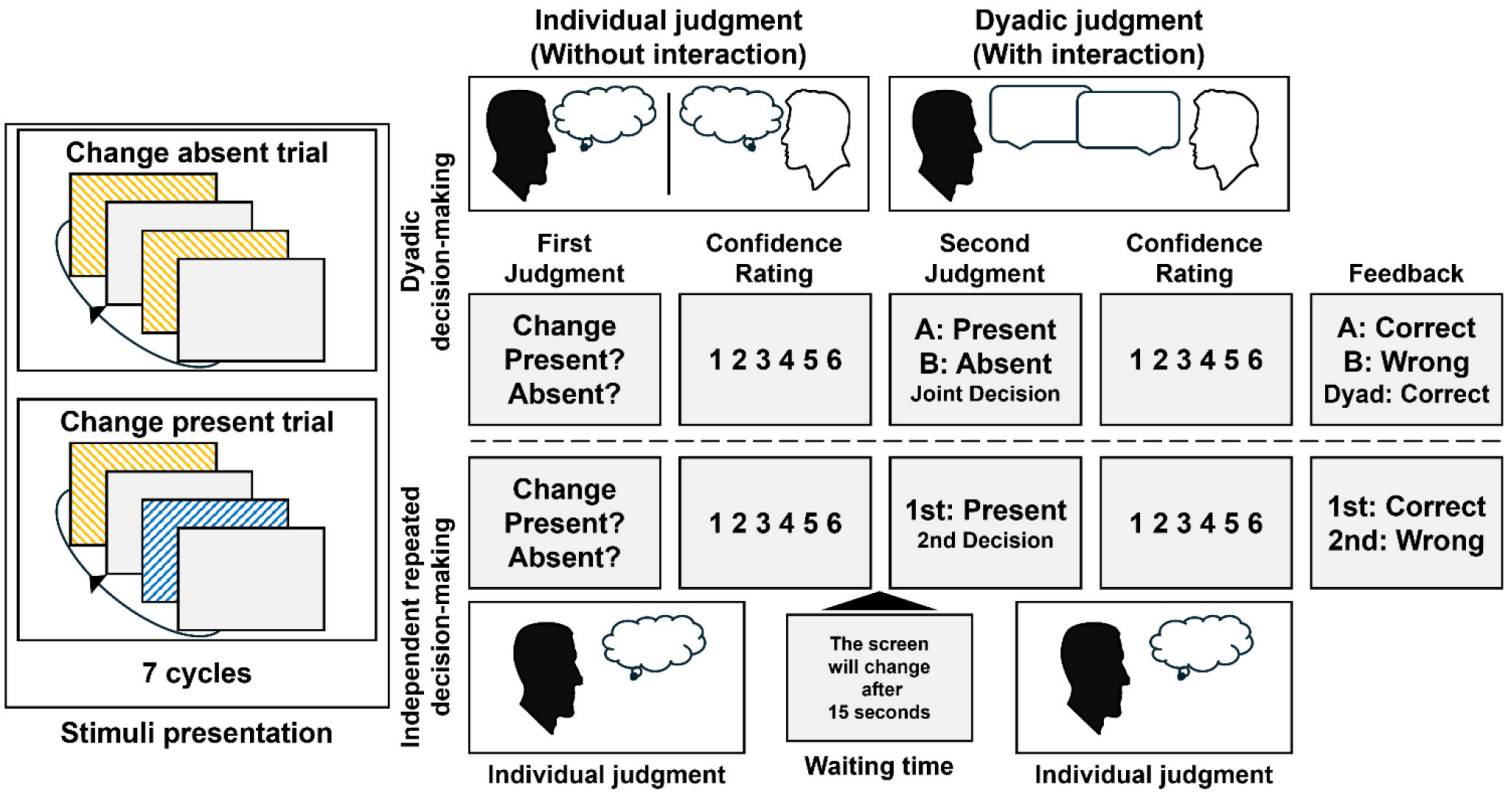
General procedures for the dyadic decision-making (upper section) and follow-up (lower section) experiments.

In the dyadic decision-making experiment, participants responded individually and collectively after the same stimulus image presentation (Bahrami et al., 2010; Kawachi and Kawachi, 2025) (Figure 1). Thus, unlike the conventional flicker paradigm (Rensink et al., 1997), in which participants judge the presence or absence of a change during the flickering sequence, we employed a modified version in which participants responded only after a preset number of flickers had been presented. In each trial, two images were presented alternately seven times, with a gray blank field between them. The number of repetitions was determined based on the selected stimuli's mean and median reaction time data (Sareen et al., 2016) to ensure comparable task difficulty across stimuli. Original and modified (or original) images were presented in the change-present (or change-absent) trial. Particularly in the change-present trial, the order of the original and modified images was randomized, and half of the change in the scene appeared on the left (or right) side of the image. Each image and gray field were presented for 250 ms (15 frames). After the presentation, each participant privately judged whether the two images had changed and rated their confidence (1 = certainly change absent to 6 = certainly change present). After the individual judgment, each participant's responses were presented on the display. The dyads communicated freely and made dyadic decisions and confidence ratings. Finally, the results of the individual and dyadic choices were displayed.

In the follow-up experiment, participants completed the task individually to test whether individual second judgment without social interaction could alter the sensitivity and decision criterion. The stimuli presentation, first decision, and confidence rating were the same as those in the dyadic decision-making experiment. After the first confidence rating, a 15-sec waiting time was set. The waiting time was determined by the approximate conversation time in dyads in the dyadic decision-making experiment. The display showed the time remaining until the screen changed. Participants were informed only that the display would change after 15 sec; they were given no other instructions to avoid experimenter bias that might help them to reconsider or alter their judgments. Following the waiting time, the first response was displayed, and participants made the second decision and confidence rating, with the option to change their initial judgments. Finally, the results of the first and second decisions were displayed. The dyadic decision-making and follow-up experiments consisted of six blocks of 16 trials (48 change-absent and 48 change-present).

### 2.5 Analysis

The measures were calculated based on SDT (Green and Swets, 1966; Stanislaw and Todorov, 1999). First, we calculated the individual and dyad judgments' hit and false alarm rates (Table 1). Some participants exhibited a false alarm rate of 0. Thus, following Macmillan and Creelman (2005), we replaced 0 with $\frac{1}{2k}$, where k is the number of change-absent trials. Second, receiver operating characteristic (ROC) analysis was conducted based on individual and dyad confidence ratings. However, most participants (data from 40 individuals and 18 dyads) failed to generate ROC plots because they did not use certain confidence ratings, indicating that the assumptions required to apply classical indices of *d*′ for sensitivity and *c* for decision criterion could not be reliably verified (Green and Swets, 1966; Stanislaw and Todorov, 1999). Therefore, we calculated *A*′ and *B*″ as the non-parametric sensitivity and decision criterion indices, respectively (Grier, 1971).

**Table 1:** Mean values of hit and false alarm rates in each condition in the dyadic decision-making (left columns) and follow-up (right columns) experiments.

Measure	Dyadic decision-making experiment		Follow-up experiment	
	Individual (*n* = 44)	Dyad (*n* = 22)	First judgment (*n* = 26)	Second judgment (*n* = 26)
Hit rate	0.532 (0.099)	0.703 (0.086)	0.563 (0.068)	0.551 (0.070)
False alarm rate	0.009 (0.019)	0.011 (0.020)	0.065 (0.076)	0.050 (0.064)

Values in brackets represent standard deviations.

For the statistical analysis in the dyadic decision-making experiment, we followed previous studies (Bahrami et al., 2010, 2013) and compared the sensitivity between ${A^{\prime }}_{better}$ and ${A^{\prime }}_{dyad}$, where ${A^{\prime }}_{better}$ refers to that of the better member in each dyad. Regarding decision criterion, we compared ${B^{\prime \prime }}_{better}$ and ${B^{\prime \prime }}_{dyad}$, where ${B^{\prime \prime }}_{better}$ refers to the decision criterion of the member with better sensitivity in each dyad. This comparison allowed us to examine whether dyads just adopted the decision criterion of the more sensitive member. Paired *t*-tests were conducted to compare ${A^{\prime }}_{better}$ with ${A^{\prime }}_{dyad}$ and ${B^{\prime \prime }}_{better}$ with ${B^{\prime \prime }}_{dyad}$, respectively.

Regarding the follow-up experiments, we computed *A*′ and *B*″ for the first and second decisions based on the hit and false alarm rates (Table 1), for the same reason as in the dyadic decision-making experiment (23 participants failed to generate ROC plots). A paired *t*-test between the first and second decisions was conducted on *A*′ and *B*″, respectively. Analyses were conducted using R (R Core Team, 2022).

## 3 Results

### 3.1 Dyadic decision-making experiment

The left columns of Table 1 present descriptive statistics for hit and false alarm rates in the dyadic decision-making experiment. As shown, the mean hit and false alarm rates for individual judgments (*n* = 44) were 0.532 (*SD* = 0.099) and 0.009 (*SD* = 0.019), respectively, whereas those for dyad judgments (*n* = 22) were 0.703 (*SD* = 0.086) and 0.011 (*SD* = 0.020).

**Figure 2:**
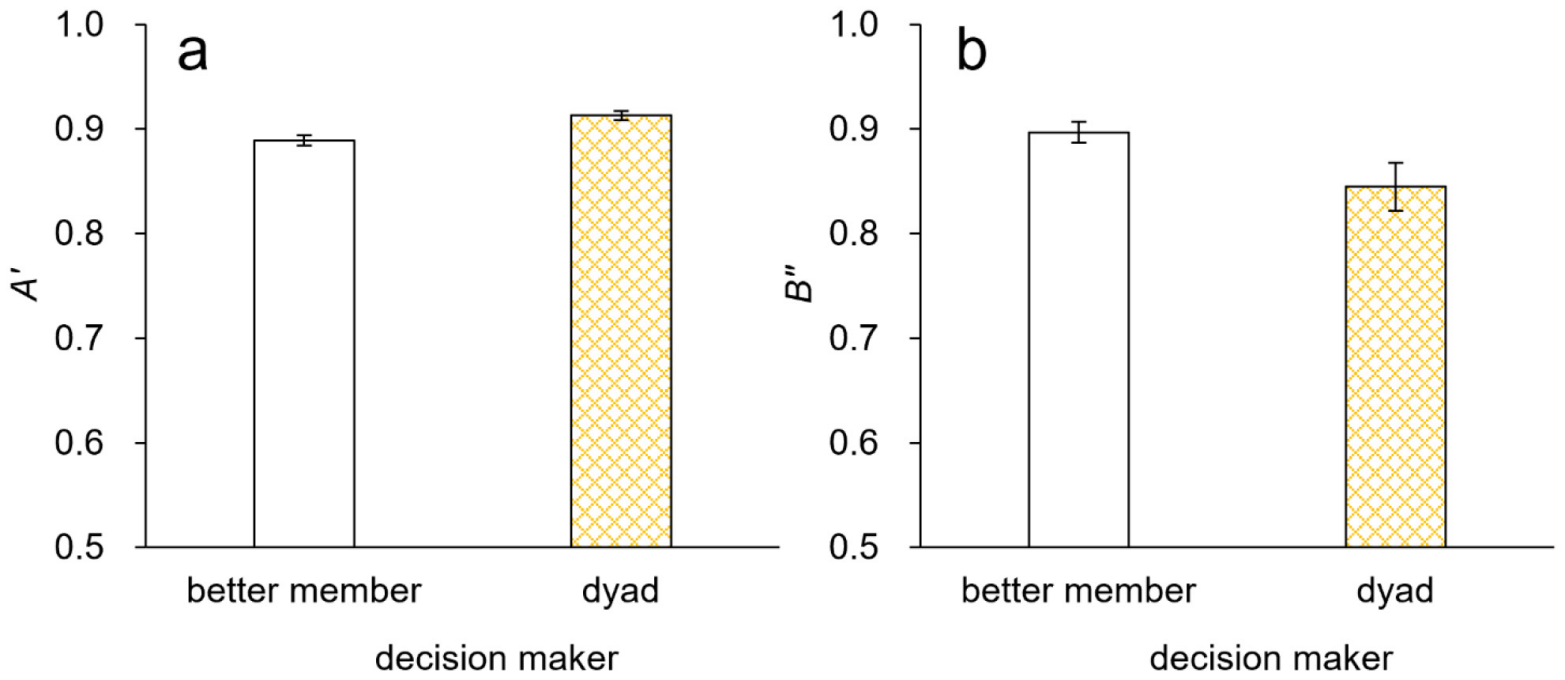
Mean **(a)** *A*′ and **(b)** *B*″ for each condition of the dyadic decision-making experiment. Error bars represent the standard error (*n* = 22).

As for the sensitivity, Figure 2a shows the mean *A*′ for each condition. A paired *t*-test showed that the dyad (*M* = 0.918, *SE* = 0.005) had significantly higher sensitivity than the better members in each dyad (*M* = 0.889, *SE* = 0.005) (*t* (21) = 13.34, *p* < 0.001, Cohen's *d* = 2.843).

Regarding the decision criteria, Figure 2b shows the mean *B*″ for each condition. A paired *t*-test showed that the decision criterion of dyads (*M* = 0.845, *SE* = 0.023) was significantly lower than that of the better members in each dyad (*M* = 0.897, *SE* = 0.010) (*t* (21) = 3.120, *p* < 0.01, Cohen's *d* = 0.665).

### 3.2 Follow-up experiment

The right columns of Table 1 present descriptive statistics for hit and false alarm rates in the follow-up experiment. As shown, the mean hit and false alarm rates for the first judgment (*n* = 26) were 0.563 (*SD* = 0.068) and 0.065 (*SD* = 0.076), respectively, whereas those for the second judgment (*n* = 26) were 0.551 (*SD* = 0.070) and 0.050 (*SD* = 0.064).

**Figure 3:**
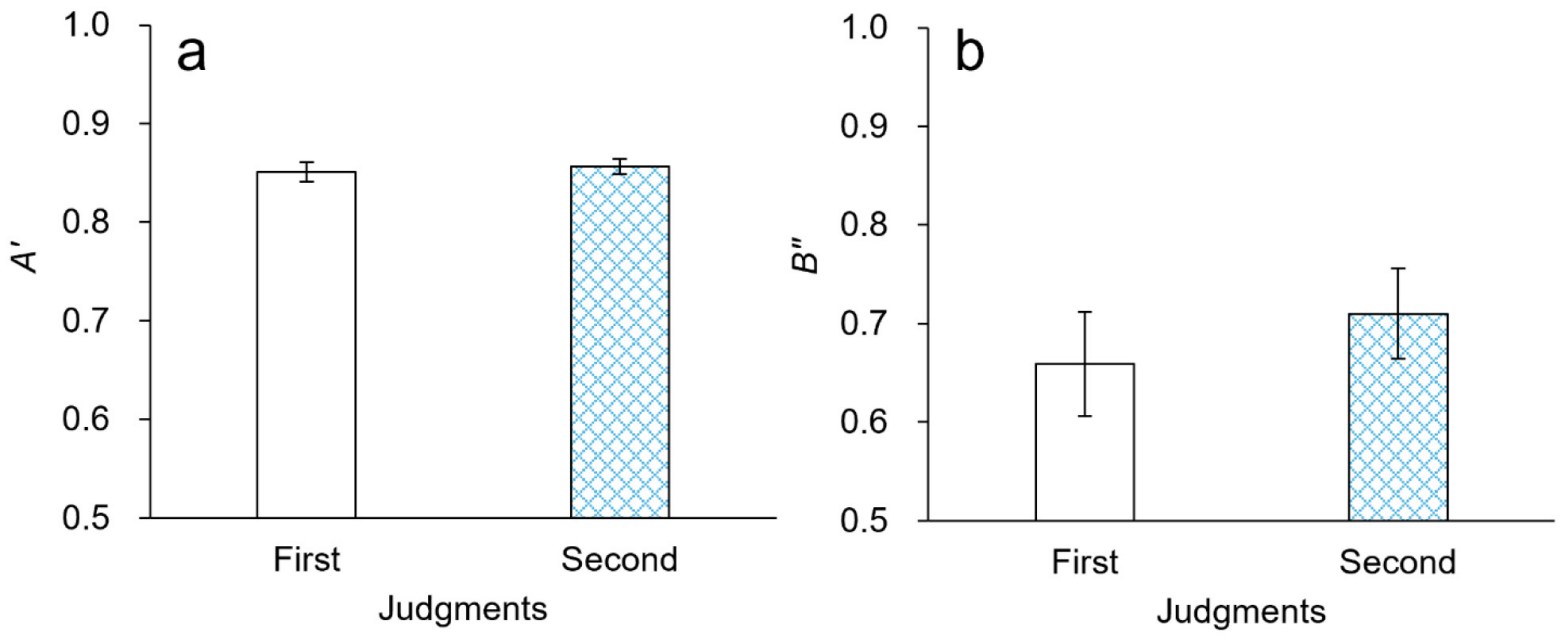
Mean **(a)** *A*′ and **(b)** *B*″ for each condition of the follow-up experiment. Error bars represent the standard error (*n* = 26).

Figure 3 shows the mean **(a)** *A*′ and **(b)** *B*″ for the first and second judgments. There were no significant differences between the first and second judgments in either the sensitivity (*A*′) (first judgment, *M* = 0.853, *SE* = 0.010; second judgment, *M* = 0.860, *SE* = 0.007) (*t*(25) = −1.443, *p* = 0.162, Cohen's *d* = 0.283) or decision criteria (*B*″) (first judgment, *M* = 0.659, *SE* = 0.061; second judgment, *M* = 0.720, *SE* = 0.050) (*t*(25) = −1.926, *p* = 0.065, Cohen's *d* = 0.378).

## 4 Discussion

This study investigated whether dyadic decision-making enhances visual sensitivity and shifts the decision criterion in a change detection task. As predicted, the dyads showed significantly greater sensitivity than the more sensitive members of each dyad, suggesting dyadic enhancement rather than dependence on better members. Moreover, enhancement of sensitivity was seen only in the dyadic judgment condition in the dyadic decision-making experiment, while the sensitivity of individuals was constant between the first and second judgments in the follow-up experiment. The same pattern was seen if the behavioral data met criteria to apply for *d*′ (see Supplementary Figures 1a and 2a). Consistent with previous findings (Bahrami et al., 2010), our results suggest that collaborative decision-making improves change detection in natural scenes. Prior research in experimental psychology has indicated that dyads outperform individuals in low-level perceptual tasks, such as contrast discrimination of Gabor patches (Bahrami et al., 2010) and dot enumeration (Bahrami et al., 2013). The current findings extend this evidence to more complex and ecologically valid tasks.

We also found that dyads adopted a more liberal decision criterion than the more sensitive members of each dyad. These results suggest that dyads adjusted their decision criterion to avoid a missed detection rather than simply relying on the one with high sensitivity. However, a criterion shift was not seen when the individuals made decisions twice in the follow-up experiment, whereas if the parametric index of *c* was used, the second judgment was more conservative than the initial judgment (see Supplementary Figures 1b and 2b). This pattern is consistent with prior work (Kawachi and Kawachi, 2025) demonstrating that criterion shifts occur during collaborative change detection with Gabor patches. In real-world situations, successful change detection depends on both perceptual sensitivity and response bias. For example, experienced drivers tend to adopt a more liberal criterion, anticipating potential hazards and responding more quickly than novice drivers despite equivalent sensitivity (Wallis and Horswill, 2007). Although research has traditionally emphasized sensitivity, our findings highlight the significance of decision criteria, echoing arguments in psychophysics regarding the influence of response bias (Peters et al., 2016). The present study shows that this factor significantly affects collaborative change detection under naturalistic conditions.

The current study provided insights into dyadic decision-making. First of all, we demonstrated that the influence of dyadic decision-making on sensitivity and decision criterion can extend not only to low-level perceptual detection (Kawachi and Kawachi, 2025) but also to higher-order detection. Also, the current study suggested a new possibility that dyadic interactions might differ depending on the task characteristics. Based on the present results and participants' introspection reports, individuals tended to hold strong confidence in their “change present” responses when they detected salient changes, regardless of their detection performance across trials. Thus, dyadic communication often led the pair to adopt the “change present” response, although they did not simply adopt a strategy where the dyad responds “change present” if either member of the dyad responds “change present” (see results of Exploratory model comparison in Supplementary material). This pattern suggests that the dyadic detection sensitivity enhancement may arise even when the two observers do not match in their individual detection sensitivity. In contrast, previous work, such as by (Bahrami et al. 2010), which used a bias-free paradigm, has shown that dyads typically improve their discriminative sensitivity only when their performance is well aligned. This is because individuals with different performance levels may show imperfect confidence communication (Bang et al., 2017). For instance, an incorrect partner may express high confidence, which can influence the final joint decision. The present findings, therefore, point to a potentially different mechanism of dyadic decision-making in a yes–no change detection task in which response bias can be measured. In this context, the dyad may reduce miscommunication that would otherwise lead to an incorrect “change absent” decision, unlike in perceptual discrimination tasks. Further research is needed to clarify the underlying mechanisms of dyadic decision-making from the perspective of task characteristics in change detection.

Previous studies (e.g., Rensink et al., 1997) have documented the change blindness phenomenon and its underlying mechanisms such as attention (e.g., Rensink et al., 1997) and visual representation (Rensink, 2002; Simons, 2000). In parallel, researchers have identified factors that modulate change detection performance, such as interest in the changing object in a scene (Rensink et al., 1997), expertise (Werner and Thies, 2000), and task demands (Lee et al., 2023). These findings indicate that change detection is shaped by top-down factors that guide attention to relevant scene information and orient observers toward particular aspects of the detection task. The current study extends this perspective by showing that dyadic decision-making can also modulate change detection performance, not only by enhancing sensitivity but also by shifting the decision criterion.

The increase in sensitivity suggests an improved ability to discriminate change-present from change-absent trials, whereas the liberal criterion shift may reduce missed detections at the possible cost of increased false alarms. This distinction between sensitivity and decision criterion also has implications for naturalistic change detection situations. In naturalistic situations, like baggage screening at an airport checkpoint, teams of security officers who are more likely to notice potential anomalies may be beneficial for preventing subsequent accidents, although this may come at the cost of increased false alarms. The current study indicated the utility of dyadic decision-making to compensate for some individual limitations in detecting changes in the scene, while we did not elucidate the underlying mechanisms of change blindness itself.

One limitation of this study is the relationship between dyadic collaboration and reaction time. Previous studies on change detection have used detection accuracy and reaction time as key performance measures (Andermane et al., 2019; Rensink et al., 1997). Similarly, a liberal criterion has been associated with faster responses, such as in the detection of potential hazards in a scene (Egea-Caparrós et al., 2016; Wallis and Horswill, 2007). In contrast, in our paradigm, dyadic reaction time could not be determined because participants first made private judgments and confidence ratings before engaging in open discussion. However, in real-world situations, rapid detection is crucial for appropriate action. Future studies should explore whether slower collaborative decisions reduce response bias, as (Dekel and Sagi 2020) suggested, thereby clarifying how dyadic discussion dynamics affect collective accuracy and bias regulation. Understanding this relationship can provide insight into the underlying mechanisms of group decision-making. Another limitation is the variation between the two members, even though dyads viewed the same images in each trial. We did not account for individual differences in perceptual sensitivity and factors such as visual stability and visual ability (Andermane et al., 2019), interest in the changing element (Rensink et al., 1997), expertise in the current context (Werner and Thies, 2000), and task demands (Lee et al., 2023), all of which may modulate dyadic performance. Investigating the effects of interpersonal dynamics and complementary skills may yield deeper insights into collective decision-making in perceptual tasks. Also, we need to consider the social aspects of dyadic interaction. Results of the follow-up experiment supported the findings in the dyadic decision-making experiment by indicating that the second judgments without social input did not differ from the initial judgments, but only to a limited extent. The results of the dyadic decision-making experiment could potentially be interpreted in terms of other interaction factors. For example, the presence of others may facilitate (Zajonc, 1965) or hinder (Latané et al., 1979) the performance without a specific interaction. In a related vein, recent studies have considered interactions with computers (Nazar et al., 2021). Mueller et al. (2024) reported that binary information from an automated face recognition system can shift the decision criterion of human participants toward “match” judgments in a face recognition task. Future research should distinctly address these factors of interaction for further understanding of dyadic decision-making.

In summary, dyadic decision-making enhanced sensitivity and shifted the decision criterion toward a more liberal position in a change detection task. These findings extend previous research on dyadic decision-making (Bahrami et al., 2010; Kawachi and Kawachi, 2025) and contribute to change detection research by showing that social decision-making can modulate both sensitivity and decision criterion. These results suggest that two-person decision-making may reduce missed changes by increasing the likelihood of reporting signals under uncertainty, while also increasing the possibility of false alarms. Although the failure of change detection is a thought-provoking phenomenon for understanding the mechanisms of visual perception and awareness (Simons and Levin, 1997; Simons and Rensink, 2005), effective ways to mitigate it remain unclear. In this context, the current study helps clarify how dyadic interaction influences perceptual sensitivity and decision criterion in a dyadic change detection paradigm. Although these findings suggest a mechanism through which two-person judgments can reduce missed changes in experimental settings, whether similar effects occur in real-world environments remains an open question for future research.

## Data Availability

The datasets presented in this study can be found in online repositories. The names of the repository/repositories and accession number(s) can be found in the article/Supplementary material.
