# Supplementary material for: Dyadic interaction enhances sensitivity and shifts decision criterion in a change detection task
Source: Front Cognit. 2026 Aug 3;5:1827978. doi: 10.3389/fcogn.2026.1827978 (PMC13477956; doi:10.3389/fcogn.2026.1827978)
Supplement: Supplementary file 1 [file Data_Sheet_1.docx]

Supplementary Material

# 1 Supplementary Analysis

Researchers often use *d’* (sensitivity) and *c* (criterion) based on the classical signal detection theory model (Green and Swets, 1966; Stanislaw and Todorov, 1999), while the current study did not apply them because our data did not meet the application criteria (normality and equal variance). Therefore, we provide supplementary information on the analysis using *d’* and *c* in this section.

## 1.1 Analysis using parametric measurements *d’* and *c*

The indices of *d’* and *c* are calculated based on the hit ($p_{Hit}$) and false alarm ($p_{FA}$) rates as follows:

*d’* = z($p_{Hit}$) - z($p_{FA}$) (1)

*c* = -0.5 (z($p_{Hit}$) + z($p_{FA}$)). (2)

We computed *d’* and *c* based on hit and false alarm rates in individual and dyad in the dyadic decision-making experiment. For the supplementary analysis, in the same manner as the main analysis, we compared ${d'}_{better}$ and ${d'}_{dyad}$, where ${d'}_{better}$refers to that of the better member in each dyad. Also, we compared $c_{better}$ and $c_{dyad}$, where $c_{better}$refers to the decision criterion of the member with better sensitivity in each dyad. Paired *t*-tests were conducted to compare ${d'}_{better}$ with ${d'}_{dyad}$ and $c_{better}$ with $c_{dyad}$, respectively.

Regarding the follow-up independent repeated decision-making experiment, we computed *d’* and *c* based on hit and false alarm rates in the first and second judgments. A paired *t*-test between the first and second decisions was conducted on $d’$ and $c$, respectively.

## 1.2 Results and Discussion of supplementary analysis

### 1.2.1 Dyadic decision-making experiment

As for the sensitivity, Supplementary Figure 1a shows the mean $d’$ for each condition. A paired *t*-test showed that the dyad (*M* = 2.717, *SE* = 0.080) had significantly higher sensitivity than the better members in each dyad (*M* = 2.468, *SE* = 0.059) (*t*(21) = 6.399, *p* < .001, Cohen’s *d* = 1.364).

Regarding the decision criteria, Supplementary Figure 1b shows the mean $c$ for each condition. A paired *t*-test showed that the decision criterion of dyads (*M* = 0.809, *SE* = 0.035) was significantly lower than that of the better members in each dyad (*M* = 1.026, *SE* = 0.028) (*t*(21) = 9.714, *p* < .001, Cohen’s *d* = 2.071).

These results supported the results in the dyadic decision-making paradigm by indicating that the performance fluctuations in dyads were robust regardless of indices.

**Supplementary Figure 1.** Mean (a) $d’$ and (b) $c$ for each condition of the dyadic decision-making experiment. Error bars represent the standard error (*n* = 22).

### 1.2.2 Follow-up experiment

As for the sensitivity, Supplementary Figure 2a shows the mean $d’$ for each condition. A paired *t*-test showed that there was no significant difference between the first (*M* = 1.875, *SE* = 0.114) and second (*M* = 1.938, *SE* = 0.100) judgments (*t*(25) = 1.485, *p* = .150, Cohen’s *d* = 0.291).

Regarding the decision criteria, Supplementary Figure 2b shows the mean $c$ for each condition. A paired *t*-test showed that the decision criterion of the second judgment (*M* = 0.838, *SE* = 0.052) was significantly higher than that of the first judgment (*M* = 0.778, *SE* = 0.058) (*t*(25) = 2.108, *p* < .05, Cohen’s *d* = 0.413).

**Supplementary Figure 2.** Mean (a) $d’$ and (b) $c$ for each condition of the follow-up experiment. Error bars represent the standard error (*n* = 26).

The result of sensitivity was consistent with that of the follow-up experiment, supporting the result where the second judgments without social interaction did not differ from the initial judgments. In contrast, the result of the decision criterion showed that the second judgments were more conservative than those of the initial judgments when participants responded twice without social input. Also, the direction of the shift in the decision criterion was opposed by that of the dyadic decision-making condition, suggesting that dyadic interactions mitigate the tendency for the decision criterion to become conservative. However, these results were valid under the assumption of classical signal detection theory (Green and Swets, 1966; Stanislaw and Todorov, 1999). Thus, they should be treated as references.

# 2 Exploratory model comparison

We compared the performance between dyads and the more sensitive members in each dyad to investigate whether the fluctuations in sensitivity and decision criterion result from the dependence on more sensitive members in each dyad. On the other hand, there are other models to compare with the dyad data. In this section, we report the exploratory model comparisons.

## 2.1 Model settings

We proposed three models: the less sensitive member dominant model, the average-of-two-members model, and the yes dominant model.

### 2.1.1 Less sensitive member model

The less sensitive member model, unlike the comparison in the dyadic decision-making experiment, assumes that the dyadic decision is made based on the dependence on less sensitive members in each dyad. In this model, the sensitivity and decision criterion are referred to ${A'}_{less sensitive}$ and ${B"}_{less sensitive}$, respectively.

### 2.1.2 Average-of-two-members model

The average-of-two-members model (Bartlett and McCarley, 2017) assumes that the dyadic performance is the average of two members by adopting the opinion of either of the two members with equal probability. In this assumption, ${Hit}_{ave}$ and ${FA}_{ave}$ are calculated as follows:

${Hit}_{ave}$ = 0.5 (${Hit}_{A}$ + ${Hit}_{B}$) (1)

${FA}_{ave}$ = 0.5 (${FA}_{A}$ + ${FA}_{B}$), (2)

where A and B are denoted as two members of each dyad. If ${FA}_{ave}$ became 0, following Macmillan and Creelman (2005), we replaced 0 with $\frac{1}{2k}$, where k is the number of change-absent trials. After that, ${A'}_{ave}$ and ${B"}_{ave}$ were computed.

### 2.1.3 Yes dominant model

The yes dominant model (Bartlett and McCarley, 2017) assumes that the dyad responds “change present” if either member responds “change present”. In this assumption, ${Hit}_{Yes dominant}$ and ${FA}_{Yes dominant}$ are calculated as follows:

${Hit}_{Yes dominant}$ = 1 – (1 - ${Hit}_{A}$)(1 - ${Hit}_{B}$) (3)

${FA}_{Yes dominant}$ = 1 – (1 - ${FA}_{A}$)(1 - ${FA}_{B}$), (4)

where A and B are denoted as two members of each dyad. If ${FA}_{Yes dominant}$ became 0, following Macmillan and Creelman (2005), we replaced 0 with $\frac{1}{2k}$, where k is the number of change-absent trials. After that, ${A'}_{Yes dominant}$ and ${B"}_{Yes dominant}$ were computed.

## 2.2 Comparison models

For each of $A’$ and $B”$, we compared the dyad and each of the three models using a paired *t*-test, respectively.

Regarding sensitivity (Supplementary Figure 3), paired *t*-tests revealed that sensitivity of actual dyad data (*M* = 0.918, *SE* = 0.005) was significantly higher than data from the less sensitive member model (*M* = 0.856, *SE* = 0.007) (*t*(21) = 17.781, *p* < .001, Cohen’s *d* = 3.791) and the average-of-two- members model (*M* = 0.873, *SE* = 0.005) (*t*(21) = 23.404, *p* < .001, Cohen’s *d* = 4.990). In contrast, $A’$ of the actual dyads was significantly lower than that of the yes dominant model (*M* = 0.935, *SE* = 0.005) (*t*(21) = -9.579, *p* < .001, Cohen’s *d* = 2.042).

**Supplementary Figure 3.** Mean $A’$ for actual dyads and each model. Error bars represent the standard error (*n* = 22). The *p*-values indicate the results from the *t*-tests between actual dyads and each model.

As for the decision criterion (Supplementary Figure 4), paired *t*-tests revealed that actual dyad data (*M* = 0.845, *SE* = 0.023) were significantly lower than the average-of-two-members model (*M* = 0.888, *SE* = 0.015) (*t*(21) = -2.703, *p* < .05, Cohen’s *d* = 0.576) while were significantly higher than that of the yes dominant model (*M* = 0.780, *SE* = 0.034) (*t*(21) = 3.255, *p* < .05, Cohen’s *d* = 0.694). However, there was no significant difference between the dyad and the less sensitive member model (*M* = 0.858, *SE* = 0.026) (*t*(21) = -0.609, *p* = .549, Cohen’s *d* = 0.130).

**Supplementary Figure 4.** Mean $B"$ for actual dyads and each model. Error bars represent the standard error (*n* = 22). The *p*-values indicate the results from the *t*-tests between actual dyads and each model.

These comparisons showed that either sensitivity or decision criterion measures were significantly different from the actual data, indicating that these models did not reflect dyadic interaction. Also, these models did not assume a particular dyadic interaction because they hold when using data from two independent members. Therefore, actual dyads did not behave like two independent observers whose responses are simply pooled under simple decision rules. Future research should propose models that can describe both sensitivity and decision criterion in dyadic decision-making.

# References

Bartlett, M. L., and McCarley, J. S. (2017). Benchmarking aided decision making in a signal detection task. *Hum. Factors* 59, 881–900. doi: 10.1177/0018720817700258

Green, D. M., & Swets, J. A. (1966). *Signal Detection Theory and Psychophysics*. New York, NY: Wiley.

Macmillan, N. A., & Creelman, C. D. (2005). *Detection Theory: A User’s Guide* (2nd ed.). Mahwah, NJ: Erlbaum.

Stanislaw, H., & Todorov, N. (1999). Calculation of signal detection theory measures. *Behav. Res. Methods Instrum. Comput.* 31, 137–149. doi: 10.3758/BF03207704
